# Self-Ligating Bracket Systems: A Comprehensive Review

**DOI:** 10.7759/cureus.44834

**Published:** 2023-09-07

**Authors:** Shalabh Baxi, Anand A Tripathi, Virag Bhatia, Mangleshwar Prasad Dubey, Pratiksha Kumar, Hiroj Bagde

**Affiliations:** 1 Department of Orthodontics, Government Dental College, Raipur, IND; 2 Department Of Orthodontics, Saraswati Dhanwantari Dental College and Hospital, Parbhani, IND; 3 Department of Orthodontics, Government College of Dentistry, Indore, IND; 4 Department of Orthodontics, Guru Gobind Singh College of Dental Sciences and Research Centre, Burhanpur, IND; 5 Department of Oral Pathology and Microbiology, Government College of Dentistry, Indore, IND; 6 Department of Periodontology, Rama Dental College and Research Centre, Kanpur, IND

**Keywords:** archwire, self-ligating brackets, orthodontics, friction, efficacy

## Abstract

Currently, ligature-free bracket technologies, including self-ligating brackets (SLBs), are all the rage in orthodontics. Self-ligating mechanisms have been shown to be more effective and less time-consuming in orthodontic treatment than traditional appliances due to their enhanced frictional properties. Crucial to the success of the multi-band/bracket method is the transmission of forces and moments from the bracket to the archwire. Advances in bracket design and ligation techniques are constantly being made to better distribute loads and increase the efficiency of leveling.

## Introduction and background

A self-ligating bracket (SLB) is defined as a bracket that utilizes a permanently installed, movable component to entrap the archwire. SLB systems represent a significant advancement in contemporary orthodontic treatment, offering a departure from traditional ligated brackets and archwire configurations. These innovative systems have garnered substantial attention and interest within the orthodontic community due to their distinct design, mechanism, and potential clinical advantages [1].

Conventional orthodontic treatment typically involves the use of elastomeric or metal ligatures to secure the archwire within the bracket slot, regulating the forces exerted on the teeth [1]. In contrast, SLB systems integrate specialized mechanisms within the bracket itself, minimizing or altogether eliminating the need for external ligatures. This distinctive approach facilitates the frictional control of tooth movement, thereby enhancing treatment efficiency and outcomes [2].

The evolution of SLB systems encompasses a wide range of design variations, from passive systems that allow for natural bracket-to-archwire engagement, to active systems that enable controlled, customized adjustments. These designs aim to optimize force delivery, minimize friction, and potentially expedite treatment duration, making them an excellent option for orthodontists and patients alike [3]. Orthodontic practitioners can gain a thorough awareness of the role of these systems in modern orthodontic treatment approaches by delving deep into their special characteristics and clinical consequences.

## Review

Development of self-ligating bracket systems

The labial face of an SLB is made of metal and may be opened and closed. Orthodontists have been using similar brackets for a surprisingly long time; in 1935, Stolzenberg described the Russell Lock edgewise attachment. The use of SLBs in orthodontics is not new but rather it was revived in the early 20th century. SLBs have been lauded for their purported benefits over traditional ones, which include quicker ligation, less friction, a shorter treatment period, fewer office visits, and less discomfort overall (higher treatment efficiency). SLB systems have been praised for their prospective benefits, the most persuasive of which include shorter treatment times and reduced subjective pain [3]. As a result of reduced bio-stability, chair-side manipulation, and periodontal health promotion are also said to be enhanced. Initial retrospective studies have shown clear benefits, including a four to seven-week reduction in total treatment duration and a corresponding drop in required sessions [1-4].

There are two basic types of SLBs: active and passive, thus named for their respective closure procedures [1]. Self-ligating active brackets store energy in a spring clip that applies pressure to the archwire to regulate rotation and torque. Active SLBs include those manufactured by Great Atlantic and Pacific Company (GAC) International (Central Islip, NY), Strite Industries (Cambridge, Ontario, Canada), and Adenta (Gilching, Munich, Germany), such as the In-Ovation, Speed, and Time, respectively. To avoid applying any active force to the archwire, passive SLBs often have a slide that may be closed without interfering with the slot lumen. SmartClip (3M Unitek, Monvoria, CA) and Damon (Ormco, Glendora, CA) are two well-known passive design products, despite SmartClip's superficial similarity to traditional brackets and its lack of sliding [2].

Classification of self-ligating brackets

The classification of SLBs is based on several aspects, including mechanism of action, material composition, and location. SLBs can be classified into passive, active, or semi-active, depending on the force application dynamics. Similarly, categorization based on the material used results in the distinction between metal and tooth-colored brackets. Moreover, the location of these brackets can be denoted as labial or lingual, reflecting their positioning relative to the tooth surface.

Brackets that are passive use a mobile, stiff component to enclose the archwire. With passive braces, tooth movement is limited only by the snugness of the archwire in its bracket slot. Inadequately sized wires contained in a structure resembling an archwire tube may affect tooth control [3], e.g., Damon, Mobil-Lock, etc.

In active brackets, the archwire is held in place in an active bracket by a bendable component. The archwire is held in place in the archwire slot by this elastic component, which may also store and release energy. With this method, the tooth and its supporting components receive a constant yet mild amount of pressure, allowing for regulated and accurate motion. Until the archwire is entirely seated in the archwire slot, i.e., the “home” position, the bracket is able to realign itself and its corresponding tooth in three dimensions thanks to the flexible component's homing action [3], e.g., Damon, Mobil-Lock, etc.

In semi-active or interactive brackets, several companies promote their films as being somewhat active or even interactive. The archwire has to grow to a specific length before the clip can release it. Before that point, the wire and clip are not really touching [3].

Self-Ligating Labial Brackets [4-7]

Russell Lock edgewise attachment: The first SLB, the Russell attachment was developed by a New York orthodontic pioneer, Dr. Jacob Stolzenberg, in the year 1935. A flat headscrew was securely fastened into a threaded circular hole on the bracket's front [4].

Edgelok brackets: In 1972, Dr.Jim Wildman of Eugene, developed the Edgelok bracket, which had a round body with a rigid labial sliding cap. To implant the archwire, the slide had to be repositioned occlusally using a specialized opening tool. By pressing down on the cap with the index finger, the archwire slot in the bracket is transformed into a tube [4].

Mobil-Lock brackets: In 1974, Dr. Franz Sander constructed a Mobil-Lock bracket. In order to open or shut a Mobil-Lock, a specialized tool was needed to spin the semicircular labial disk. The archwire was passively enclosed inside a tube produced by the outside wall of the bracket slot.

Speed system: Hanson [5,6] combined his own concept for a strong, self-ligating machine with that of Points' edgewise machine. The end result is a non-ligature design that keeps the curved wire contained inside the curved wire aperture and under effective control with the help of a spring-stacked, self-changing instrument. Strite Industries Ltd. of Ontario, Canada has commercialized this idea into production as the Speed appliance.

Speed appliance design: The four parts that make up a Speed attachment are the bracket body, the spring clip that is permanently attached to the bracket, the in-out adapter, and the foil mesh bonding base. Speed attachments seem identical but are really custom-made for each individual tooth [5].

The bracket body: The speed appliance has a slim, one-bracket design. The precisely machined bracket body has many holes for various components, including a slot for the archwire that has already been torqued, an auxiliary slot, and a spring retention slot [4]. Archwires of any form or size may be inserted into the archwire slot, which measures either 0.018 x 0.025 inches or 0.022 x 0.028 inches.

The spring clip: The Speed bracket may be identified by its roll-shaped, flexible spring clip. In lieu of the steel or elastomeric tie used for ligation in conventional brackets, this vertically opening and closing spring clip makes removing and inserting archwires a breeze.

The in-out adaptor: This remarkable quality allows the development of an incredibly smooth arch shape by using a gradual “ramp-like” effect of the in-out adaptors. With the new, more robust design of each adapter, manufacturers have been able to reduce the size of their appliances while simultaneously boosting the bond strength by more than 300%.

The mesh pad: Each Speed fastener has a bonding pad made of foil mesh with intricate asymmetric curves. These, together with the reduced size of each mesh pad, allow for perfect tooth-bracket adaptability.

Auxiliaries for Speed System

The Speed bracket system was designed with a horizontal auxiliary hole measuring 0.016 x 0.016 inches to improve archwire dynamics and permit the use of detachable bracket hooks. Uses of this parallel slot include the placement of a 0.016-inch Supercable as a secondary archwire to assist in the alignment of severely displaced teeth, such as lingually maligned lateral incisors, or to assist in the eruption of impacted canines while the main archwire continues to stabilize the occlusion [6].

Archwires: The Speed bracket is an edgewise appliance that accepts edgewise archwires and permits the use of conventional archwire mechanics [6].

Supercable archwires: A seven-stranded, super-elastic, nickel-titanium coaxial wire, “Supercable” was developed to work in tandem with the nickel-titanium spring clip. Unmatched as an initial aligning and leveling wire, Supercable exerts dramatically reduced force levels compared with solid nickel-titanium and thermally sensitive wires of similar dimensions. The periodontium will stay close to ideal across the whole deactivation spectrum [5].

D-Wire: D-wire is a one-of-a-kind hybrid of round and square wire that excels at three-dimensional control in sliding mechanics. Both 0.018 x 0.018-inch and 0.021 x 0.021-inch D-Wire are on hand for your wiring needs.

Hills Dual-Geometry archwire: The polished, round posterior portion of the Hills Dual-Geometry archwire facilitates efficient sliding mechanics in the posterior segments, while the wire's square anterior segment ensures precise torque control over the incisor crowns. There are two different sizing options for the anterior portion of the Hills archwire: 0.018 x 0.01 8 inches with a 0.018-inch round posterior for the 0.018 slot, and 0.021 x 0.021 inches with a 0.020-inch round posterior for the 0.022 slot [8].

Activa Brackets

The labial surface of the Activa bracket slot was concave because of the spinning slide (“A” Company, San Diego, CA). Since the effective slot depth was raised, labiolingual alignment was decreased while using wires with short diameters. Retaining the slide on the mesial and distal ends of the slot resulted in a broader-than-usual bracket, which in turn decreased the interbracket span and its benefits. 

Self-ligation may be advantageous since it allows for the use of shorter sections, which leads to a longer interbracket length, which in turn results in lower powers and a wider range of activity with any particular archwire during the planning stage [9-14].

Self-ligating interactive bracket system: Initial therapy involves passively low levels of force and friction. Active torque and rotational control are present throughout the course of therapy. There are few contacts in and out of the circle. It is characterized by a clip mechanism that opens and closes easily for changing wires, and the ability to precisely apply finishing touches in any of the three spatial dimensions.

The bonding base and twin body of the bracket are both cast (metal injection molded) from a single piece of metal. A “comma”-shaped stainless steel retention clip is oriented on the facial surface and “hung” from the gingival tie wings of the bracket body.

Time 2 (American Orthodontics)

The base and body of the Time 2 bracket are manufactured as a single unit utilizing the metal injection molding (MIM) process, making it an active system. By inserting a specific instrument labially, the bracket may be opened and the mechanism can be hinged gingivally. A specialized instrument is used to secure the bracket [5].

Advantages: The Time 2 bracket may be set up with little effort and does not need specialized training. It can be opened and closed quickly and easily, and the indications are clear. Depending on how securely the bracket locks into place, it may provide impressive rotational control.

Disadvantages: The large size of the bracket makes it unattractive. The closing mechanism becomes jammed up with elastomeric chains. Rotational and torque control may be challenging since the locking mechanism does not always transfer the forces of the clip to the archwire. A steel ligature might be helpful in certain situations.

Indications: The Time 2 bracket is a substantial metal appliance that allows for the ligation of archwires with diameters more than 0.020.

Contraindications: Patients with tiny teeth, allergies to the metal components, or strict aesthetic standards should look elsewhere. It might be difficult to keep a rotation under control [12].

Time 3 (American Orthodontics)

When compared to the Time 2 bracket, the Time 3 bracket is much more compact. MIM manufacturing is used for both the bracket's base and body.

Advantages: The Time 3 bracket's opening and closing mechanisms are intuitive and need little practice to master. It is simple to recognize the symbols.

Disadvantages: The locking mechanism might be compromised by elastomeric chains. In many cases, the clip's tension is insufficient to provide maximum torque and rotational control.

Indications: Lignation of very thick rectangular archwires (more than 0.020) is possible with the Time 3 bracket since it is a sturdy medium-sized bracket. Newcomers to the field may benefit from reading it.

Contraindications: High esthetic standards or known allergies to the alloy components of the bracket rule out the use of this bracket for these individuals [5].

Damon Appliance System

The Damon system, introduced in the mid-1990s, had a slide that encircled the bracket's labial face. The theory is predicated on the idea that the threshold force must be applied in order to commence tooth movement [15]. The Damon SLB was designed to satisfy the following major criteria: Andrews Straight wire appliance concept, Twin configuration, Slide forming a complete tube, Passive slide on the outside face of the bracket, and brackets opening inferiorly in both arches.

The pre-set Damon appliance may be purchased with either a 0.022-inch or 0.018-inch opening. Metal injection molding is used to create Damon tubes because it allows for the production of very precise, miniature components, such as those responsible for the slide's freedom of motion and the archwire slot's tight tolerances.

Bracket design: After its first release as the Damon SL bracket, the Damon bracket design settled into its current iteration, which has the following characteristics: Unlike active self-ligating designs, this one is passive and uses regular tie wings; a gate that opens to reveal the slot and self-ligates using a positive mechanism to keep it open or closed. These characteristics have altered as the bracket has developed: Patients will find this new, slimmer bracket with its lower profile and softer, rounder edges more to their liking. A vertical auxiliary slot is included on the D3 MX bracket [5].

Damon standard prescription: The Damon standard prescription is recommended for all molars and premolars, all incisors and canines in good position, and labially inclined canines.

Damon 2 brackets (Ormco Corporation): Damon 2 brackets were introduced to address the imperfections of Damon SL. They still used the same U-shaped spring and vertical slide motion to regulate the opening and shutting, but the slide is now concealed by tie-wings.

Damon 3 brackets (Ormco Corporation): The holding spring of Damon 3 brackets was placed and operated differently, enabling a smooth, reliable opening and closing operation.

Damon 3 brackets are also rather aesthetically pleasing. However, there were three major issues with the first batch of Damon 3 production brackets: a high rate of bond failure, metal separation from the reinforced resin components, and cracked resin tie-wings. Both the all-metal Damon D3 MX bracket released more recently, and the succeeding Damon Q bracket released thereafter obviously benefitted from the lessons learned from earlier production challenges and from further clinical experience and competence [5].

The Damon Q and Q2 brackets represent major advancements in orthodontic technology. These brackets are part of a bracket system used in orthodontic treatments. The Damon Q and Q2 brackets are designed to improve the alignment of teeth and the overall effectiveness of orthodontic procedures. The key feature of these brackets is their self-ligating mechanism, which means that they have a built-in mechanism to hold the archwire in place without the need for additional elastic or metal ligatures. This design can lead to reduced friction, potentially allowing teeth to move more freely and comfortably. The design of these brackets takes into account lessons learned from previous models and clinical experiences, leading to improvements in their performance [15].

Twin Lock Bracket

The Twin lock bracket was unveiled in 1998. The flat, rectangular slide is forced occlusally into the empty area using a large scaler, and then it is trapped between the tie-wings of an edgewise twin portion. When the device slides gingivally in response to finger pressure, the archwire is stuck in a neutral position [16].

Disadvantage: Its commercial success has faded because of the slide's lack of mobility while opening and shutting.

Oyster Self-Ligating Bracket

Translucent SLBs first appeared on the market in 2003. The objective was to provide a cosmetically preferable alternative to conventional composite brackets and self-ligating metal brackets. The composite polymer and fiberglass used to make the bracket ensure its durability. Polycarbonate (PC) makes up 70% of the Oyster ESL, while polyethylene terephthalate (PET) accounts for the remaining 30%. The bracket is a standard or Roth prescription edgewise bracket with a .018 or .022 slot [16].

Disadvantages: Poor dimensional stability and staining nature.

In-Ovation R (GAC)

In-Ovation brackets were introduced in the market in the year 2000 by GAC Company. The In-Ovation R has an electronic bracket. The body, which harkens back to the traditional twin design, rests on a well-delineated and sculpted bracket [17].

Advantages: The In-Ovation R bracket is simple to install, can be opened and closed quickly, and provides superior control over rotation and torque. Stretchy chains may be positioned either on top of or underneath archwires.

Disadvantages: Since it is made of metal, this bracket is not aesthetically pleasing [17]. Bracket opening and closure issues have also been linked to archwire diameters of 0.018, in the 0.022 system and higher.

Indications: By using In-Ovation R brackets, even extreme crowding may be easily remedied. With rectangular archwires (bigger than 0.020), torque may be managed well.

Contraindications: Allergy to chromium-molybdenum or nickel precludes the use of the In-Ovation bracket [5].

In-Ovation C (GAC)

The In-Ovation C is a smart piece of pottery. The section base and the twin body are combined into a single piece via fired infusion forming. The recording's matte quality is perfect from an aesthetic standpoint. Comparatively speaking, the metal In-Ovation R portion is smaller than its plastic version. This area may be accessed from the gingival course for all teeth except the molars and lower premolars utilizing a specially designed device. The firm also provides ceramic tubes for attaching the lower premolars. However, when bigger archwires are inserted or removed, these tubes often crack [6, 18].

Advantages: This bracket outperforms the In-Ovation R bracket in terms of adjusting rotations and transmitting torque values, and it is just as simple to place and open and shut. It is simple to add elastomeric chains over or under the archwires. When relocating brackets, it might be helpful to be able to reuse the bracket (for the same patient) after cleaning the bracket base with a sandblaster, as the manufacturer states is feasible owing to the robust manufacturing process.

Disadvantages: The clip's plastic construction makes it inferior to the metal In-Ovation R version. Bracket debonding may be difficult owing to the size of the bracket base and the bracket material, and chromium-molybdenum alloys are difficult to rhodium-plate.

Indications: Good torque control is achieved thanks to tooth movement in big rectangular wires (>0.020).

Opal Bracket

It is a sort of a passive bracket. Translucent fiber-reinforced composite polymer is its main component. It is made in one seamless piece, and the lid includes a built-in mechanism that allows it to self-ligate.

Advantages: The Opal bracket is quite attractive since it is incredibly smooth and soothing on the sensitive tissues. It can be placed accurately and has clear, legible markings.

Disadvantages: Loss of a bracket is quite frequent. The placement of elastomeric chains and the opening of this bracket also present challenges [6].

Indications: For shorter-term treatment plans, Opal brackets may be recommended. Correcting minor rotations and crowding is straightforward. In its early stages, this bracket is aesthetically pleasing, making it a good option for patients with strict aesthetic needs.

Opal M (Ultradent)

The MIM method is used to create the passive Opal M bracket.

Advantages: The delicate tissues will not be irritated by the Opal M bracket. It is just as simple to set in place as its more aesthetically pleasing version thanks to markings on the lid's outside. A new design makes it simple to place elastic chains in any desired location.

Disadvantages: The mechanism's opening may be difficult. Also, the bracket's design and surface qualities make it seem bigger than it really is.

Indications: Patients with very delicate soft tissues may benefit from using this bracket. This passive bracket is ideal for rotational and torque management of the teeth and may be used for any form of malocclusion because of its size and design.

Contraindications: Extreme crowding makes this a bad idea. The metallic surface of the bracket makes it seem quite dark in the patient's mouth; therefore it may not be aesthetically pleasing enough for certain people [19].

SmartClip Bracket System

SmartClip SLBs were introduced by 3M Unitek Company in the year 2005. These are passive SLBs with McLaughlin Bennett Trevisi (MBT) prescriptions. These brackets are twin brackets that engage the wire using a nickel-titanium clip. The nickel-titanium alloy from which this clip is made gives it a remarkable ability to retain its form after being bent or twisted [8,9].

The SmartClip Self-Ligating Appliance System was developed with the same goals of versatility, bracket prescription, and little manipulation as the MBT Versatile+ Appliance System. There is no way to open or shut the bracket since there is no latch or door. Because there are no moving parts, such as doors or latches, these brackets are less likely to have issues like sticking, spontaneous opening, plaque buildup, etc. than other kinds of SLBs. SmartClip brackets are the only true SLBs, closing and securing the archwire in the wire slot automatically [10,20].

Engagement and Disengagement of Wires

Engagement: A rectangular notch at one end of the working key facilitates archwire engagement in orthodontic treatment. This notch may be used by professionals to gently guide the archwire into the bracket slot, where it can be secured behind the clips with little force. While applying force to the wire, the clinician should support the tooth using his or her fingers on the lingual side of the mouth [20].

Disengagement: Orthodontic archwires may be extracted from the bracket slot by rotating the functional key in the opposite direction. The disengagement tool's central component has two hooks to make contact with the wire, and it is supported by the buccal surface of the mesial and distal wings. The wire may be unhooked from the slot in the bracket with a simple spin [21].

Quick 2 (Forestadent)

The Quick bracket is an operational bracket. It is made in a single piece using MIM and sintering technology. The chromium-molybdenum alloy is used to create the clip's elastic material.

Advantages: The Quick bracket may be set up quickly and easily. The markings on the bracket make finding your way around much simpler, and the clip mechanism may be deployed with little effort. It provides excellent rotational and torque control, with simple elastomeric chain insertion. Archwires with diameters of 0.021 and greater may be ligated. It also has an auxiliary slot measuring 0.016 x 0.016.

Disadvantages: This metal bracket, like other metal brackets, may not live up to the aesthetic expectations of certain patients.

Indications: This is a compact, sturdy metal bracket for use in cases of extreme congestion when rotational control is of the utmost importance [11,12].

Contraindications: These are mostly cosmetic, but also include the possibility of an allergy to the metals used to make the bracket.

Clarity SL (3M Unitek)

The ceramic body of the Clarity SL bracket makes it a passive system. The frictional properties of the ceramic base are enhanced by a metal groove [13,14].

Advantages: The placement of the Clarity SL bracket is identical to that of the conventional ligation version. Both the rotational and torque controls work well. Elastomeric chains may be quickly and readily installed thanks to the bracket's obvious indications. The patient can easily keep it clean since there are no moving components, such as lids or other locking devices. Because ceramic brackets are designed with a deliberate weak area that breaks when the correct debonding procedure is used, they are comparatively easy to remove.

Disadvantages: Pain is often experienced by patients during the affixing and unfastening of highly hard and hefty archwires. It is possible that the tooth-colored coating on esthetic archwires may chip during ligation. Due to its intentional weak spot in the vertical axis, this bracket may only be removed after orthodontic treatment is complete and cannot be used again for realignment or repair.

Indications: The main benefits of this bracket are its speed and accuracy in leveling and alignment, as well as its specified frictional qualities. Most of the metal components are concealed by the archwire, making it aesthetically beautiful as well.

Contraindications: Since removing the archwire and performing the ligation may be painful, particularly for the lower incisors, this bracket is not advised for individuals with a high threshold for pain. Because the clip has the potential to harm the archwire's esthetic coating during ligation, it is also not suggested to utilize first-generation esthetically coated archwires. The hybrid wires used for bigger diameters fall short of ordinary rectangular wires with sharp edges, which might be a problem in cases requiring a lot of torque [13].

Vision LP (American Orthodontics)

The Vision Low Profile (LP) is a dynamic, tie-winged system. MIM sintering is used to create the frame and base.

Advantages: It is simple to set up the Vision LP brackets. They may debond somewhat more readily than other SLBs, especially in the lower jaw, due to the larger thickness of the brackets. Inconspicuous markings and a smooth hinge make this bracket a breeze to use. 

Disadvantages: Sometimes, the tension applied by the locking mechanism to the archwire is insufficient for complete torque and rotational control. Steel ligatures, which may be wrapped around the tie-wings, are a possible aid. 

Indications: The Vision LP bracket is a robust metal bracket of medium size that facilitates the simple ligation of very heavy archwires (greater than 0.020 in diameter).

Contraindications: The cavity in the locking mechanism is fairly big, making it easy for food particles and plaque to get lodged beneath the archwire [22].

Discovery SL (Dentaurum)

Passive in design, the Discovery SL bracket has tie-wings and a curved base. The MIM sintering method is used to create the bracket's base and body.

Advantages: The base's clear markings and well-rounded shape make placing and bonding a breeze. Its ultrasmooth exterior is gentle on sensitive skin. For a self-ligating metal bracket, it has decent aesthetic attributes.

Disadvantages: Due to the seeming diminutive size of the mechanism, training is required before the bracket may be opened and closed. Because of the mesiodistal width of the bracket and the opening orientation of the door, rotational and torque control are not always optimal.

Indications: The Discovery SL system is a self-ligating bracket that can accommodate massive rectangular archwires (more than 0.020 in diameter) despite its compact size.

Contraindications: If a patient has an allergy to any of the metals used to make the brackets, they should not use them. The wire might get disengaged if the hinge doors open. If the wires are selected too early in the treatment process or if the rotations are too extreme, they will be overly huge. This is analogous to the issue reported with the Time bracket [19].

Self-Ligating Lingual Brackets

Philippe 2D (Forestadent Bernhard Förster GmbH): The brackets for the repair of mild malocclusions such as crowding or spacing, the lingual method was recommended because of the two-dimensional control it provides. These brackets do not have a slot but rather feature little wings soldered to the bottom. These low-profile brackets reduce discomfort for the patient. The medium twin (often used for the lingual technique), the large twin, the three-wing bracket for connecting intermaxillary elastics, and the narrow singlewing bracket for lower incisors are the four main types of Philippe brackets available [8,23].

Forestadent 3D torque-lingual SLB: These braces are similar to the flat, self-ligating Philippe 2D brackets, but they contain a vertical slit that allows for control in all three dimensions. With the slot oriented vertically, installing an archwire has never been simpler. The flatness of the bracket and the placement of the archwire on the tooth surface like a ribbon contribute to the bracket's low profile, and the buccolingual slot size is less than the occlusogingival slot dimension [24,25].

Adenta Evolution lingual bracket (Adenta GmbH): With the Adenta Evolution lingual bracket, the archwire may be inserted from the occlusal side thanks to the clip's design, which opens at the incisal edge. When biting, the clip may double as a bite plate, forcing the archwire further into the slot [25]. The Adenta Evolution brackets are positioned indirectly using a modified laboratory HIRO system and a custom “Smart Jig.”

In-Ovation - L (GAC International): These lingual brackets are a pair of horizontal slot brackets that have a clip that can be opened with a simple touch. The wings and clips of the brackets are extremely thin, and the brackets for the incisors have a curved base to accommodate the palatal surface of the teeth. More space may be created for the archwire and between brackets when using low-profile brackets with narrow buccolingual profiles.

Phantom (Gestenco International): This polyceramic bracket does not need additional ligation. The teeth's lingual surfaces are reshaped and any defects are filled with a flowable composite before the brackets are glued in place [23-30].

Advantage: Although these benefits apply in theory to all SLBs, the ability of various brands to offer them consistently varies [26]. This offers more certain full archwire engagement, low friction between bracket and archwire, less need for chair-side assistance, and faster archwire removal and ligation.

A summary of the pros and cons of the aforementioned SLB systems is presented in Table 1.

**Table 1:** Comparison of various self-ligating bracket systems

Bracket system	Pros	Cons
SmartClip	The self-ligating mechanism reduces friction; easy engagement and disengagement	May not meet aesthetic expectations
Discovery SL	Smooth surface for patient comfort; simple placement and bonding	Rotational and torque control may not be optimal
In-Ovation R	Good torque control; quick and easy ligation	Not aesthetically pleasing for some patients
Opal bracket	Aesthetically pleasing and comfortable	Prone to bracket loss and challenges in elastomeric chain placement
Time 2 and 3	Quick setup and opening/closing; offers good rotational control	Larger bracket size and not aesthetically appealing for some
Damon appliance	Slide mechanism for better control and passive system with low friction	Initial discomfort during engagement and disengagement
Clarity SL	Ceramic material for aesthetics and good torque control	Potentially painful affixing and unfastening of heavy archwires
Quick 2	Quick setup and good rotational control	Not aesthetically pleasing
Vision LP	Sturdy metal bracket for extreme cases and easy setup	Clip tension may not provide complete torque and rotational control
Adenta Evolution	Archwire insertion from the occlusal side	Requires specialized laboratory system for placement
In-Ovation L	Easy to open and close with touch	Thin wings may cause bending and breakage
Phantom	No need for additional ligation	Requires reshaping of teeth and additional work before placement
Philippe 2D and 3D	Low-profile brackets for patient comfort and good control in two or three dimensions	Requires specialized techniques for placement and indirect bonding

Full archwire engagement, less friction between the bracket and archwire, and faster archwire removal and ligation are just a few of the benefits offered by SLBs. However, there are a few issues with SLBs that might prevent them from being widely used. Firstly, is it more likely that a flexing clip may be damaged, distorted, or have opening/closing mistakes? This topic has not been thoroughly investigated. To test such assumptions, researchers need to conduct studies in which a wide variety of SLBs are used, either on the same patient or in a sample of patients chosen at random. Secondly, in addition to increasing the risk of occlusal interferences and lip pain, the issue pertaining to SLBs' greater profile owing to their complex mechanical construction needs to be addressed. While SLBs are now widely available, they are more costly than most high-quality tie-wing brackets.

## Conclusions

The idea of SLBs was first developed in the 1930s. They've had a renaissance in the last 30 years because of the introduction of several new appliances. Light pressures on a low-friction base are the foundation of SLB systems, guaranteeing more physiologic tooth movement and more harmonious oral interaction. 

These systems have risen in popularity in recent years due to the many benefits they are said to have over traditional appliance systems. These benefits include shorter treatment times, less subjective discomfort, better periodontal health, greater torque expression, and better arch dimensional change. Further benefits include potentially preserving the anchoring, increasing expansion, decreasing the proclination of front teeth, reducing the need for extractions, and improving infection management. Changes in appointment frequency, mechanics of treatment, archwires, wire sequences, and overall expertise level are all examples of the advantages. However, because of their enthusiasm for the new product, the practitioner may do ''a little more'' than is really required, which might cause the observer to be unfairly biased.
